# Clinicopathologic significance of *MYD88* L265P mutation in diffuse large B-cell lymphoma: a meta-analysis

**DOI:** 10.1038/s41598-017-01998-5

**Published:** 2017-05-11

**Authors:** Ju-Han Lee, Hoiseon Jeong, Jung-Woo Choi, HwaEun Oh, Young-Sik Kim

**Affiliations:** Department of Pathology, Korea University Ansan Hospital, Ansan, Republic of Korea

## Abstract

The precise clinicopathologic significance of *myeloid differentiation primary response gene* (*MYD88)* L265P mutation in diffuse large B-cell lymphomas (DLBCLs) remains elusive. To investigate the frequency and clinicopathologic significance of the *MYD88* L265P mutation in DLBCLs, we conducted a meta-analysis of 40 published studies on 2736 DLBCL patients. We collected relevant published research findings identified using the PubMed and Embase databases. The effect sizes of outcome parameters were calculated using a random-effects model. In this meta-analysis, the *MYD88* L265P mutation in DLBCL showed a significant difference according to tumor sites. The overall incidence of the *MYD88* L265P mutation in DLBCLs, excluding the central nervous system and testicular DLBCLs, was 16.5%. Notably, the *MYD88* L265P mutation rates of CNS and testicular DLBCL patients were 60% and 77%, respectively. Interestingly, the *MYD88* L265P mutation was more frequently detected in activated B-cell-like (ABC) or non-germinal center B-cell-like (GCB) than GCB subtype (OR = 3.414, p < 0.001). The *MYD88* L265P mutation was significantly associated with old age and poor overall survival, but not with sex and clinical stage. This pooled analysis demonstrates that the *MYD88* L265P mutation is significantly associated with the tumor sites and molecular subtypes in DLBCL patients.

## Introduction

Myeloid differentiation primary response gene (MYD88) is an adaptor protein that activates the nuclear transcription factor κB (NF-κB) signaling through most of the Toll-like receptors (TLRs)^1^. An L265P mutation, a change from leucine (CTC) to proline (CCG), in the MYD88 Toll/interleukin (IL)-1 receptor domain, recruits MYD88 protien to the cytoplasmic tail of TLRs to form an active complex. The complex promotes NF-κB and Janus kinase-signal transducer and activator of transcription 3 (JAK-STAT3) signaling^1^.

Recently, many investigators have reported that the prevalence of *MYD88* L265P mutation ranges from 0% to 94% in different series of diffuse large B-cell lymphoma (DLBCL) patients^2–41^. However, since the *MYD88* L265P mutation occurs at various frequencies of DLBCL, a general consensus on clinicopathologic implications has not been reached. DLBCL is a heterogeneous non-Hodgkin’s lymphoma mainly comprising molecular subtypes such as germinal center B-cell-like (GCB) and activated B-cell-like (ABC) types^42^. Several studies have suggested that the frequency of *MYD88* L265P mutation may vary depending on the tumor site or molecular subtype of DLBCL^2, 8, 21, 24, 30, 41^, but individual studies with different designs hinder clear conclusions. Furthermore, the clinicopathologic significance of the *MYD88* L265P mutation in each DLBCL patient was controversial.

To address these controversies, we conducted a meta-analysis to examine the frequency of *MYD88* L265P mutation and the relationship between this mutation and the clinicopathologic parameters of DLBCL patients.

## Results

### Prevalence of *MYD88* L265P mutation in diffuse large B-cell lymphoma

On pooled analysis of 40 studies, including 2736 DLBCL patients, the overall prevalence rate of *MYD88* L265P mutation was 29.0% [95% confidence interval (CI): 17.2–44.5%] (Table 1)^2–41^. Twenty-nine studies have reported that the frequency of *MYD88* L265P mutation in 2285 DLBCL patients except for central nervous system (CNS) and testicular lymphomas was 16.5% (95% CI: 11.9–22.6%)^2, 3, 6, 7, 9–11, 13, 16–22, 24, 25, 27, 28, 30, 31, 33–39, 41^. Thirteen^4, 5, 8, 9, 12, 14, 15, 18, 21, 23, 26, 32, 40^ and four^8, 9, 21, 29^ studies reported the prevalence of *MYD88* L265P mutation in 378 CNS and 88 testicular DLBCL patients. The *MYD88* L265P mutation in the CNS and testis were detected in 59.8% (95% CI: 42.2–75.2%) and 77.1% (95% CI: 67.1–84.7%), respectively. The prevalence of *MYD88* L265P mutation in CNS and testicular DLBCL was significantly higher than that of DLBCL in other sites (p < 0.001, Q = 49.671, *I* ^*2*^ = 95.974). As a result of subgroup analysis, the prevalence of *MYD88* L265P mutation in DLBCL patients was not significantly different according to the race (Supplementary Table S1).

**Table 1:** Characteristics of individual studies of *MYD88* L265P mutation in diffuse large B-cell lymphoma patients.

Study	Country	Ethnicity	Method	Location	L265P mutation (%)
Bohers E	France	Caucasian	Seq	LN	18/161 (11%)
Bonzheim I	Germany, UK	Caucasian	Seq	Vitreoretinal	19/28 (68%)
Braggio E	USA	Caucasian	ExomeSeq	CNS	9/14 (64%)
Bruno A	France	Caucasian	ExomeSeq, Seq	CNS	14/37 (38%)
Caner V	Turkey	Asian	Seq	LN	8/43 (19%)
Cani AK	USA	Caucasian	ExomeSeq	Orbital, ocular	3/7 (43%)
Chapuy B	USA, Germany, Netherlands	Caucasian	ExomeSeq, Seq	CNS, testes	71/104 (68%)
Choi JW	Korea	Asian	Seq	LN, CNS, testes	8/124 (6%)
Cox MC	Italy	Caucasian	AS-PCR	LN	0/17 (0%)
Fernandez-Rodriguez C	Spain	Caucasian	AS-PCR	LN	17/175 (10%)
Fukumura K	Japan	Asian	ExomeSeq, Seq	CNS	35/41 (85%)
Gebauer N	Germany	Caucasian	Seq	EBV associated	0/26 (0%)
Gonzalez-Aguilar A	France	Caucasian	ExomeSeq, Seq	CNS	11/29 (38%)
Hattori K	Japan	Asian	Targeted Seq	CNS	28/42 (67%)
Jimenez C	Spain	Caucasian	AS-PCR	LN	9/89 (10%)
Juskevicius D	Switzerland	Caucasian	Targeted Seq	LN	7/39 (18%)
Kim Y	Korea	Asian	Seq	LN, CNS	31/161 (19%)
Knief J	Germany	Caucasian	Seq	Thyroid	0/21 (0%)
Koens L	Netherlands	Caucasian	AS-PCR, Seq	Skin	4/10 (40%)
Kraan W	Netherlands	Caucasian	AS-PCR, Seq	LN, CNS, testes	38/177 (21%)
Lohr JG	USA	Caucasian	ExomeSeq	LN	3/49 (6%)
Montesinos-Rongen M	Germany	Caucasian	Seq	CNS	5/14 (36%)
Morin RD	Canada	Caucasian	RNA Seq	LN	6/96 (6%)
Nagakita K	Japan	Asian	Seq	Gastrointestinal	3/49 (6%)
Nakamura T	Japan	Asian	Seq	CNS	50/71 (70%)
Ngo VN	USA	Caucasian	Seq	LN	56/242 (23%)
Ogura G	Japan	Asian	Seq	LN	3/8 (38%)
Oishi N	Japan	Asian	AS-PCR, Seq	testes	18/22 (82%)
Pasqualucci L	USA	Caucasian	ExomeSeq, Seq	LN	8/111 (7%)
Pham-Ledard A	France	Caucasian	AS-PCR, Seq	Skin	34/58 (59%)
Poulain S	Spain	Caucasian	AS-PCR, Seq	CNS	12/23 (52%)
Raja H	USA	Caucasian	AS-PCR	Vitroretinal	14/17 (82%)
Rovira J	Spain	Caucasian	AS-PCR, Seq	LN	39/213 (18%)
Santos Gda C	Canada	Caucasian	Seq	LN	1/21 (5%)
Staiger AM	Germany	Caucasian	AS-PCR	LN	13/119 (11%)
Taniguchi K	Japan	Asian	AS-PCR, Seq	Breast	16/28 (57%)
Wang CZ	China	Asian	HRMA, Seq	LN	11/120 (9%)
Xue D	China	Asian	HRMA, Seq	LN	4/42 (10%)
Yamada S	Japan	Asian	Seq	CNS	17/18 (94%)
Zhang J	USA	Caucasian	ExomeSeq	LN	9/73 (12%)

Seq; sequencing, AS-PCR; allele-specific polymerase chain reaction, HRMA; high resolution melting analysis, LN; lymph node, CNS; central nervous system, EBV; Epstein-Barr virus.

### Relationship between *MYD88* L265P mutation and clinical parameters of DLBCL

In this study, we analyzed the relationship between *MYD88* L265P mutation and the clinical features of DLBCL patients except for CNS DLBCL and primary cutaneous DLBCL, leg type, because these two subtypes are clinically different from other DLBCL patients.

**Figure 1:**
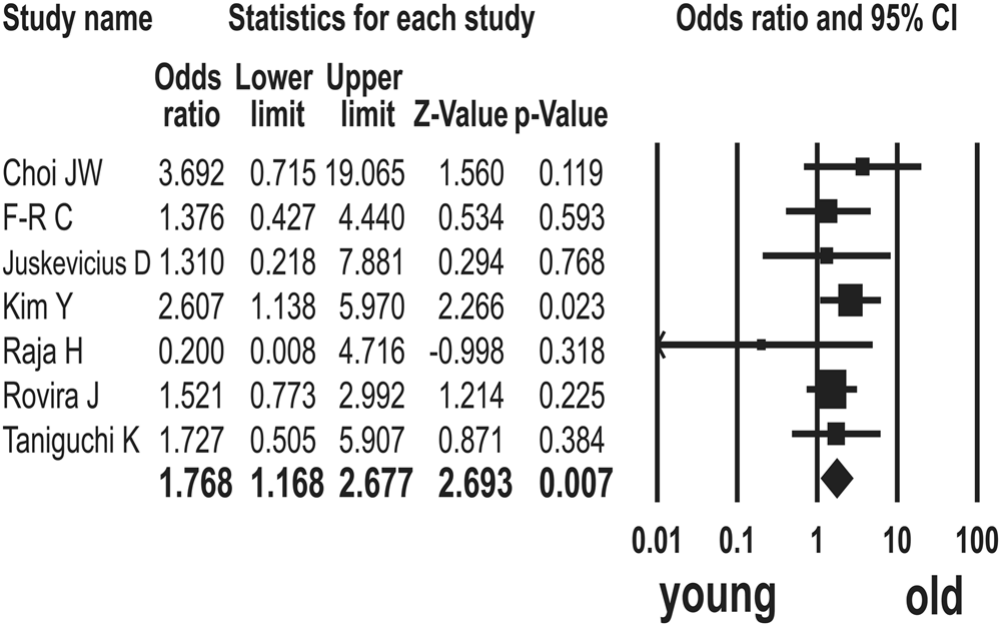
Odds ratios (ORs) with corresponding 95% confidence intervals (CIs) of individual studies and pooled data for the association between *MYD88* L265P mutation and old age. The forest plot demonstrates each study and overall effect sizes and 95% CIs.

Seven studies have described the association of *MYD88* L265P mutation with age^9, 11, 17, 18, 33, 34, 37^. The *MYD88* L265P mutation was detected in 96 (21%) of 452 patients of more than 60 years and in 43 (13%) of 320 patients of 60 years or less. The *MYD88* L265P mutation was significantly related to older age (odds ratio (OR) = 1.768; 95% CI: 1.168–2.677; p = 0.007, Q = 3.918, *I* ^*2*^ = 0.000) (Fig. 1).

Six studies presented the association between *MYD88* L265P mutation and patient’s sex^9, 11, 17, 18, 33, 34^. The *MYD88* L265P mutation was found in 69 (17%) of 411 male and in 35 (11%) of 315 female patients. No association was found between the *MYD88* L265P mutation and sex (OR = 1.566; 95% CI: 0.996–2.464; p = 0.052, Q = 4.288, *I* ^*2*^ = 0.000).

**Figure 2:**
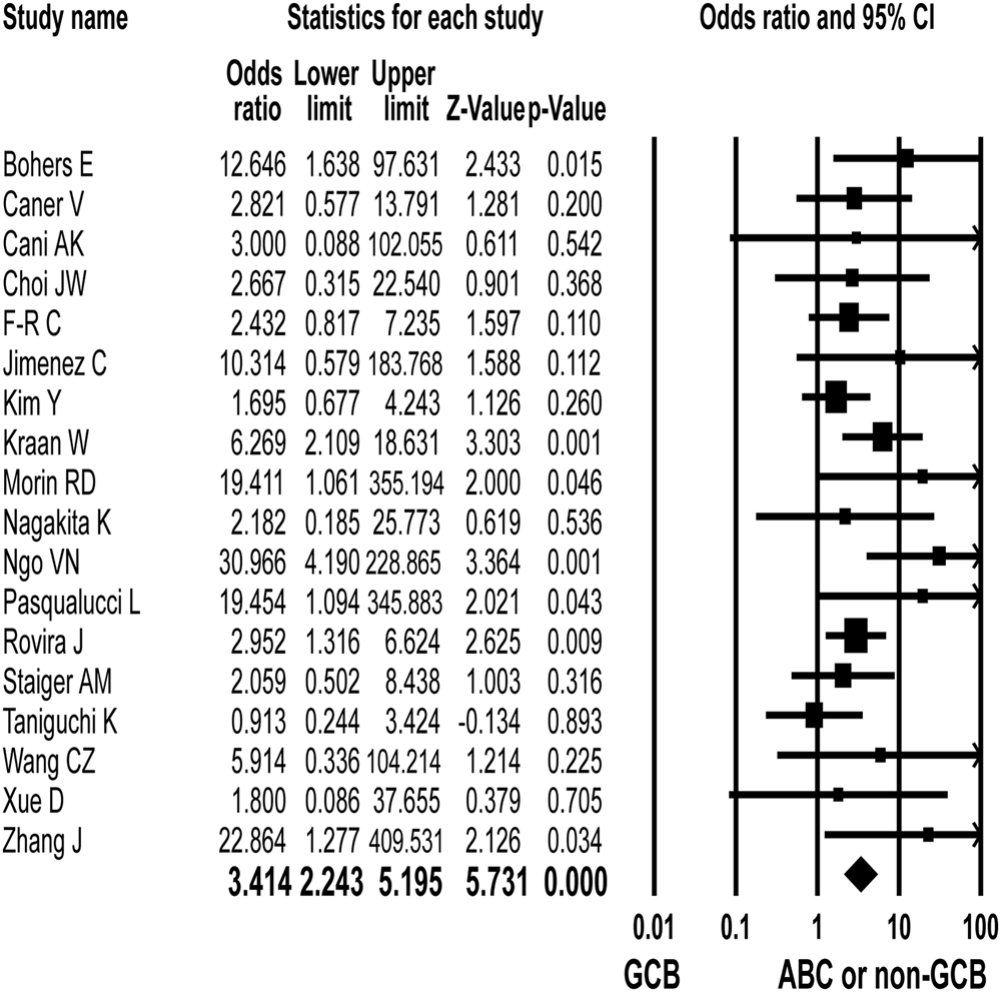
Pooled estimates of the association between *MYD88* L265P mutation and activated B-cell-like (ABC) or non-germinal center B-cell-like (non-GCB) subtype of diffuse large B-cell lymphoma. The forest plot demonstrates each study and overall effect sizes and 95% CIs.

Eighteen studies reported that *MYD88* L265P mutation was detected in 255 (21%) of 1236 activated B-cell-like (ABC) or non-germinal center B-cell-like (non-GCB) subtype and in 44 (6%) of 766 GCB subtype patients^2, 6, 7, 9, 11, 16, 18, 21, 24, 25, 27, 30, 34, 36–39, 41^. The *MYD88* L265P mutation was significantly associated with ABC or non-GCB subtype (OR = 3.414; 95% CI: 2.243–5.195; p < 0.001, Q = 19.986, *I* ^*2*^ = 14.865) (Fig. 2).

Six studies addressed the relationship between *MYD88* L265P mutation and clinical stage (III, IV versus I, II)^9, 11, 17, 18, 34, 37^. The *MYD88* L265P mutation was detected in 59 (18%) of 336 high-stage and in 101 (22%) of 465 low-stage cases. There was no association between the *MYD88* L265P mutation and clinical stage (OR = 1.244; 95% CI: 0.804–1.924; p = 0.327, Q = 2.377, *I* ^*2*^ = 0.000).

Four studies described the association between *MYD88* L265P mutation and international prognostic index (IPI) risk group^9, 11, 18, 34^. The *MYD88* L265P mutation was found in 39 (17%) of 233 cases with the high-risk group and in 54 (13%) of 427 cases with the low-risk group. No relationship was seen between the *MYD88* L265P mutation and IPI risk group (OR = 1.522; 95% CI: 0.939–2446; p = 0.088; Q = 2.883, *I* ^*2*^ = 0.000). However, sensitivity analysis revealed that Kim *et al*.^18^ study affected the pooled OR. Therefore, in the case of excluding the study by Kim *et al*.^18^, the *MYD88* L265P mutation was significantly related to high IPI risk group (OR = 1.828; 95% CI: 1.061–3.147; p = 0.030).

### Survival analysis

Four studies showed accurate multivariate hazard ratios (HRs) and CIs in 436 DLBCL patients including CNS DLBCL cases (Table 2)^11, 15, 18, 31^. In this meta-analysis, the estimated adjusted HRs ranged from 0.57 to 3.448. No association was found between the *MYD88* L265P mutation and overall survival outcomes (HR = 2.029; 95% CI: 0.873–4.713; p = 0.100, Q = 10.389, *I* ^*2*^ = 71.125).

**Table 2:** Hazard ratios and covariates used in the multivariate survival model.

Study	Case (No.)	HR (95% CI)	Variables
Fernandez-Rodriguez C	175	3.448 (1.583–7.509)	*MYD88* L265P mutation,* Hans’ phenotype, extranodal site, IPI*
Hattori K	42	2.903 (1.013–8.323)	*MYD88* L265P mutation,* age, altered mental activity,* CrCl, deep brain involvement
Kim Y	161	0.57 (0.23–1.41)	*MYD88* L265P mutation, age, IPI,* *CD79B* mutation, GI origin, chemotherapy regimen
Pham-Ledard A	58	2.94 (1.18–7.30)	*MYD88* L265P mutation,* age, multifocality,* location

HR; hazard ratio, CI; confidence interval, IPI; international prognostic index, GI; gastrointestinal *; independent prognostic variables, P < 0.05, CrCl; creatinine clearance.

**Figure 3:**
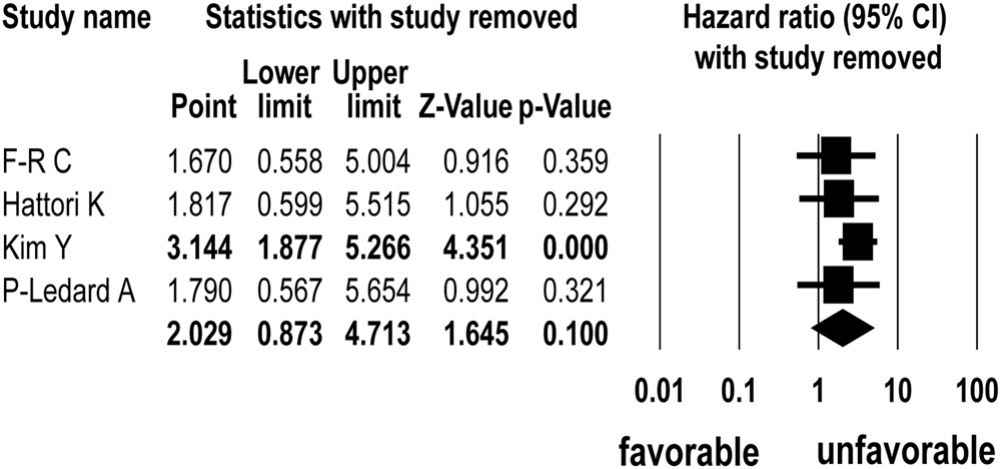
Sensitivity analysis of meta-analysis for the association between *MYD88* L265P mutation and survival outcomes.

However, we reassessed the relationship between *MYD88* L265P mutation and overall survival of DLBCL in detail based on the subgroup and sensitivity analyses. As a first step, the subgroup analysis revealed that the tumor site of origin (CNS vs. non-CNS DLBCL) and ethnicity did not affect the overall survival outcomes in patients with DLBCL (Supplementary Table S2). Although the tumor location did not affect the overall survival of DLBCL, we reanalysed the effect of *MYD88* L265P mutation on the overall survival outcomes of 394 DLBCL patients after excluding CNS DLBCL cases^15^. Nevertheless, there was no association between the *MYD88* L265P mutation and overall survival outcomes (HR = 1.817; 95% CI: 0.599–5.515; p = 0.292, Q = 9.906, *I* ^*2*^ = 79.810). Finally, the sensitivity analysis showed that Kim *et al*.^18^ study influenced the pooled HR (Fig. 3), so the meta-analysis was performed again except for Kim *et al*.^18^ study. This pooling analysis showed that DLBCL patients with the *MYD88* L265P mutation had low overall survival rates (HR = 3.244; 95% CI: 1.784–5.826; p < 0.001, Q = 0.068, *I* ^*2*^ = 0.000) (Supplementary Fig. S1).

### Sensitivity analysis and publication bias

**Figure 4:**
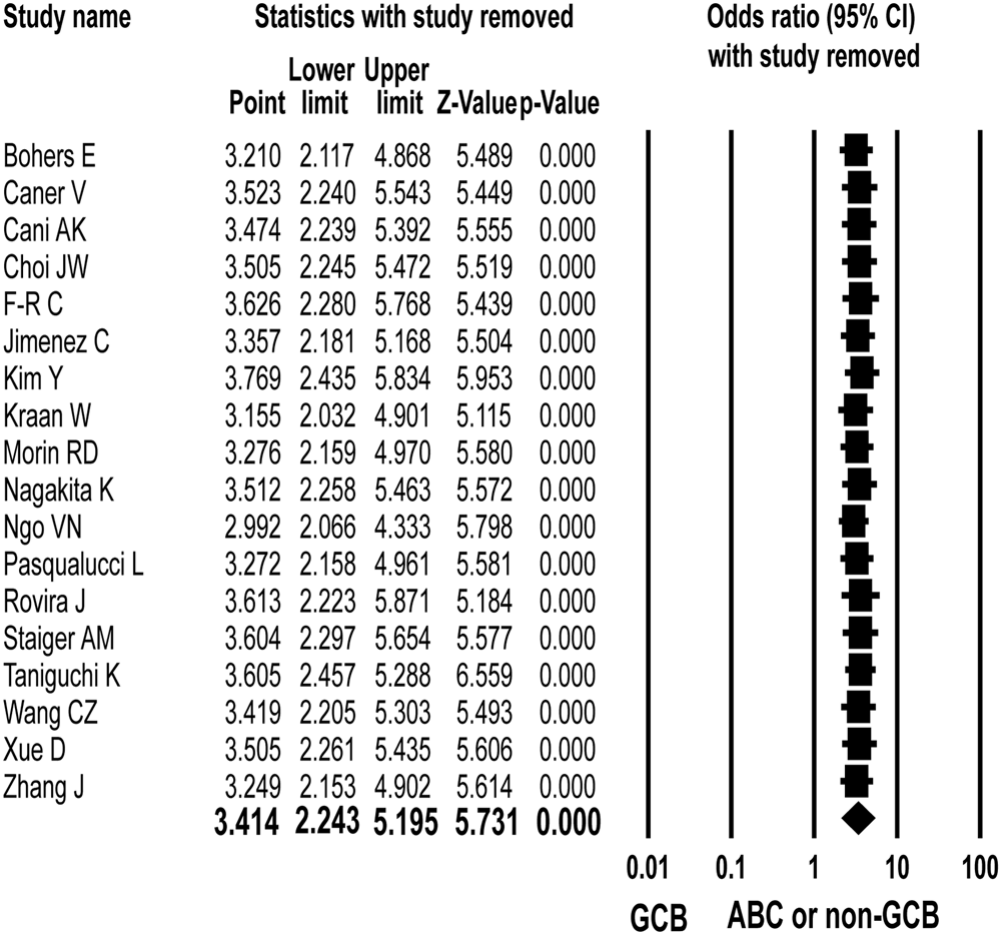
Sensitivity analysis of meta-analysis for the association between *MYD88* L265P mutation and ABC or non-GCB subtype of diffuse large B-cell lymphoma. When each study is sequentially removed and meta-analysis is repeated with the remaining studies, the pooled odds ratios (ORs) remain almost the same.

The sensitivity analysis showed that the pooled analyses of age^18^, sex^17, 34^, IPI^18^ and survival outcome^18^ according to *MYD88* L256P mutation affected the pooled OR and HR, but the other analyses revealed that none of the studies affected the pooled OR with CIs (Fig. 4).

**Figure 5:**
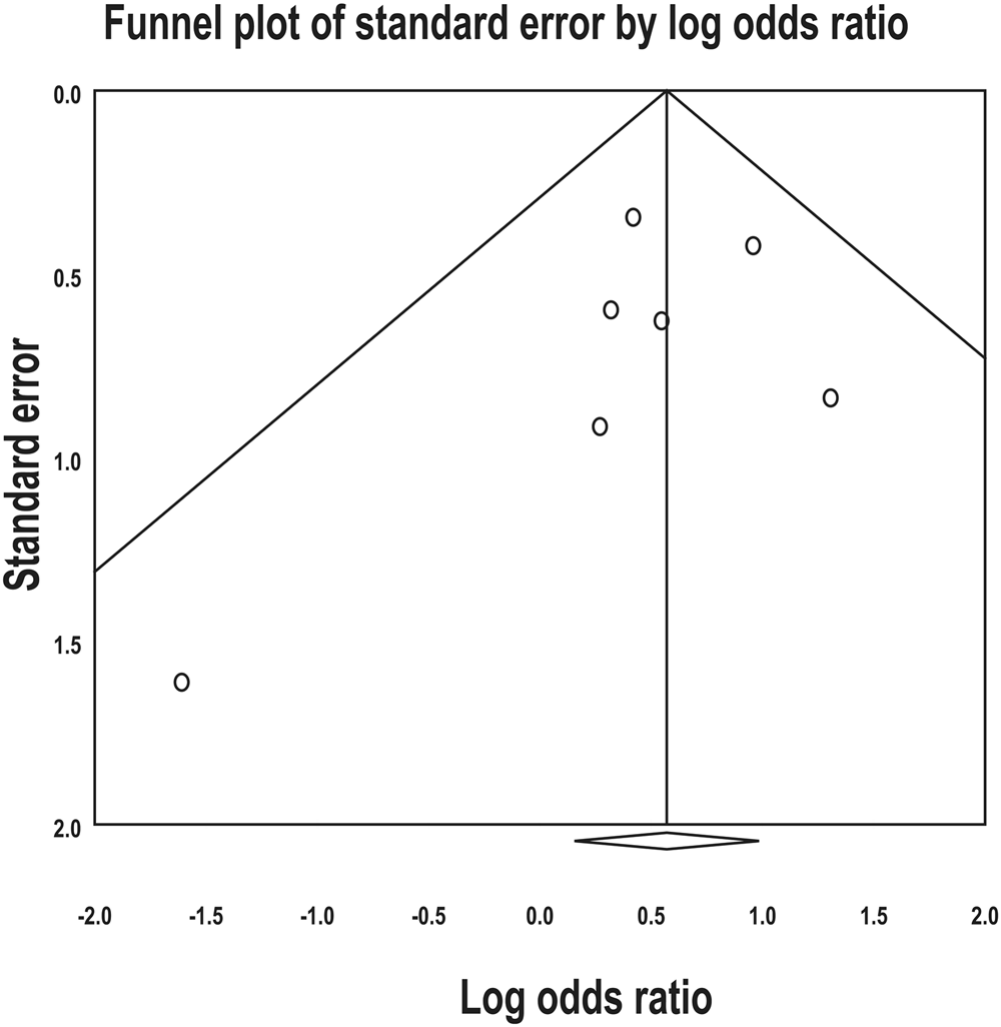
Funnel plot of meta-analysis for the association between *MYD88* L265P mutation and age. Individual studies, indicated by small circles, are uniformly distributed in inverted V-shape and indicate no published bias.

In the funnel plot and Egger regression test, there was no evidence of publication bias except for the meta-analysis for the association of *MYD88* L265P mutation with the DLBCL subtypes (Supplementary Table S3) (Fig. 5).

## Discussion

This pooled analysis showed that the tumor location of origin in DLBCL contributes to the prevalence difference of *MYD88* L265P mutation. Moreover, the *MYD88* L265P mutation is found in the ABC or non-GCB subtype more frequently than the GCB subtype of DLBCL.

We found that the incidence of *MYD88* L265P mutation in DLBCL patients varies from tumor site to tumor site. CNS and testicular DLBCLs are present as extra-nodal localized masses and show inferior responses to current chemotherapy regimens^8^. Interestingly, Kraan *et al*. suggested that the *MYD88* L265P mutation in DLBCL is associated with an immune-privileged anatomical compartment, such as the CNS or the testis^21^. Consistent with the previous result, this study showed that the *MYD88* L265P mutation is most common in DLBCL at immunologically privileged sites.

This pooled analysis revealed that the *MYD88* L265P mutation in DLBCL is significantly associated with the ABC or non-GCB subtype. ABC type DLBCL is characterized by chronic active B-cell receptor signaling and intrinsic activation of the NF-κB pathway, which can contribute to poor response to chemotherapy^43^. Initially, Ngo *et al*. claimed that GCB subtype has almost no *MYD88* L265P mutation^27^. Since then, some studies have reported that the *MYD88* L265P mutation occurs at a significantly higher frequency in ABC or non-GCB subtypes^2, 21, 24, 30, 41^. In contrast, other studies did not show a significant association between the *MYD88* L265P mutation and the DLBCL molecular subtypes^16, 18, 38^.

The prognostic value of the *MYD88* L265P mutation has been the matter of controversy. Some studies have reported that *MYD88* L265P mutation is significantly associated with low survival rates^11, 15, 31, 34^. However, we did not reach a clear conclusion through the initial pooled analysis. Except for individual studies found not to be suitable for meta-analysis by sensitivity analysis, a second pool analysis revealed that the *MYD88* L265P mutation was associated with a low survival rate and a high IPI risk group. Our results indicate that the *MYD88* L265P mutation is significantly associated with patients older than 60 years, but not with gender and clinical stage. More studies are needed to demonstrate the negative prognostic effect of the *MYD88* L265P mutation in DLBCL patients.

Some studies have mentioned other *MYD88* mutations, such as S243N and S219C other than L265P^2, 7, 21, 24, 27, 30, 34^. In particular, Rovira *et al*. compared the clinical characteristics between L265P mutation and other *MYD88* mutations^34^. However, there was not enough published data to perform a meta-analysis on this issue.

Targeting the MYD88 pathway in patients with DLBCL who do not respond well to anti-CD20 antibody therapy (rituximab) may be an attractive option. Interestingly, ABC type DLBCL with the *MYD88* mutation often responds to Ibrutinib, a selective inhibitor of Bruton tyrosine kinase (BTK)^44^. Thus, the *MYD88* mutation status of DLBCL may be a good predictor of chemotherapy in DLBCL patients.

There are several limitations to the current meta-analysis. First, we classified patients as white and Asian, but there may be discrepancies between our classification and the original data. Second, individual studies used different analytical methods and heterogeneous clinical samples. Third, the criteria for determining the molecular subtype of DLBCL differed somewhat between studies. Finally, the meta-analysis for overall survival could not be performed using the survival data from DLBCL patients completely excluding CNS and primary cutaneous DLBCL, leg type. We could not extract the survival HRs and CIs from nodal DLBCL from the published data because the previous studies presented only the survival HRs and CIs of DLBCL cases including some CNS and/or primary cutaneous DLBCLs. These limitations might affect the results of this pooled analysis.

## Conclusion

This meta-analysis indicates that the *MYD88* L265P mutation is a significant mutation in the DLBCL of the immune-privileged region and is significantly associated with the ABC or the non-GCB subtype of DLBCL. This mutation can be a powerful driver of high NF-κB activity, a characteristic of DLBCL’s ABC or non-GCB subtypes.

## Methods

### Data collection and selection criteria

**Figure 6:**
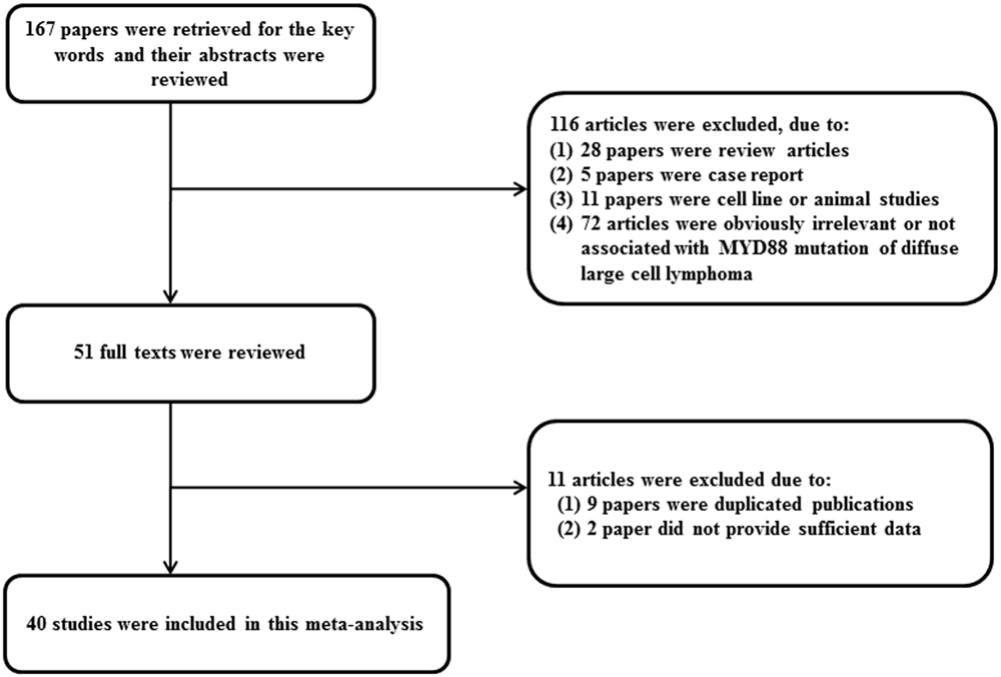
Article selection flow chart.

We searched PubMed (http://www.ncbi.nlm.nih.gov/pubmed) and EMBASE (www.embase.com) using the keywords “MYD88”, “lymphoma”, and “whole sequencing”. We also manually searched the reference lists of the identified articles. Duplicate data or overlapping articles were excluded by examining the authors’ names and affiliations. Original articles reporting cases of *MYD88* L265P mutation published before September 2016 were included. When multiple articles were published by the same authors or institutions, the most recent or single informative article was selected. Articles lacking clinicopathologic data for meta-analysis, review articles without original data, conference abstracts, case reports, and articles that dealt with cell line or animal were excluded. There were no geographic or language restrictions. The selection process of the articles is shown in Fig. 6.

### Data pooling and statistics

Meta-analysis was performed as previously described^45^. Briefly, effect sizes for each study were calculated by the prevalence rate and OR or HR and the corresponding 95% CI using the Mantel-Haenszel method or the Cohen method. The prevalence rates or ORs or HRs were combined using the random-effects model (DerSimonian-Laird method). The prevalence rates, ORs, or HRs were combined using the random-effects model (DerSimonian-Laird method). Statistical heterogeneity among studies was evaluated using the Cochrane Q test and *I* ^*2*^ statistics. The *I* ^*2*^ statistic refers to the percentage of variation across studies that is due to heterogeneity rather than chance and does not inherently depend on the number of studies considered [*I* ^*2*^ = 100% × (Q - df)/Q]. We clarified the cutoff of *I* ^*2*^ statistics for assignment of low (<25%), moderate (25–50%), and high (>50%) heterogeneities. If the *I* ^*2*^ value was more than 25%, subgroup analysis was done. Sensitivity analyses were performed to examine the influence of each study on the pooled prevalence rate and OR or HR by serially omitting an individual study and pooling the remaining studies. Publication bias was examined by funnel plots and Egger’s tests for the degree of asymmetry. If the P value is less than 0.1, it is assumed that a publication bias exists. The pooled analysis was performed using Comprehensive Meta-analysis Software version 2.0 (Biostat, Englewood, NJ, USA).

## Electronic supplementary material

Supplementary Information
