# Supplementary material for: Clinicopathologic significance of MYD88 L265P mutation in diffuse large B-cell lymphoma: a meta-analysis
Source: Sci Rep. 2017 May 11;7:1785. doi: 10.1038/s41598-017-01998-5 (PMC5431939; doi:10.1038/s41598-017-01998-5)

Clinicopathologic significance of *MYD88* L265P mutation in diffuse large B-cell lymphoma: a meta-analysis

Ju-Han Lee, Hoiseon Jeong, Jung-Woo Choi, HwaEun Oh, Young-Sik Kim

Department of Pathology, Korea University Ansan Hospital, Ansan, Republic of Korea.

Supplementary Table S1. Prevalence of *MYD88* L265P mutation in diffuse large B-cell lymphoma by race

	Category	Study (No.)	Case (No.)	Prevalence (%) (95% CI)	P value
Overall DLBCL		40	2736	29.0 (17.2 – 44.5)	
	Caucasian	27	1967	23.1 (15.5 – 33.0)	0.110
	Asian	13	769	37.7 (23.1 – 54.9)	
DLBCL excluding CNS		29	2285	16.5 (11.9 – 22.6)	
and testicular lymphoma	Caucasian	21	1715	16.8 (11.3 – 24.3)	0.880
	Asian	8	570	15.9 (8.5 – 27.9)	
CNS lymphoma		13	378	58.6 (47.7 – 68.7)	
	Caucasian	7	192	51.6 (37.9 – 65.1)	0.111
	Asian	6	186	68.9 (52.3 – 81.8)	
Testicular lymphoma		4	88	77.1 (67.1 – 84.7)	
	Caucasian	2	63	76.1 (64.1 – 85.1)	0.722
	Asian	2	25	79.7 (59.4 – 91.3)	

No.; number, CI; confidence interval, DLBCL; diffuse large B-cell lymphoma, CNS; central nervous system

Supplementary Table S2. Subgroup analysis of survival outcomes according to ethnicity and tumor site.

	Category	Study (No.)	Case (No.)	Odds ratio (95% CI)	P value
Overall survival outcome		4	436	2.029 (0.873 – 4.713)	0.100
Ethnicity	Caucasian	2	233	3.198 (1.151 – 8.882)	0.205
	Asian	2	203	1.224 (0.416 – 3.603)	
Tumor site	Lymph node	3	394	1.817 (0.599 – 5.515)	0.690
	CNS	1	42	2.903 (0.387 – 21.752)	

No.; number, CI; confidence interval, CNS; central nervous system

Supplementary Table S3. Egger's test for funnel plot asymmetry

Individual analysis	Regression intercept	P value*
Prevalence	0.338	0.789
Age (> 60 vs. 60 ≤)	-0.678	0.419
Sex	0.860	0.406
ABC/ non-GCB vs. GCB subtype	1.150	0.044
Clinical stage (III/ IV vs. I/ II)	0.777	0.397
IPI risk group	0.394	0.885
Multivariate survival outcome	-2.651	0.825

*; Two-sided P value < 0.1 was statistically significant, ABC; activated B cell-like, GCB; germinal center B-cell-like

Supplementary Figure S3. Forest plot of hazard ratios and corresponding 95% confidence intervals for the association of *MYD88* L265P mutation with unfavorable overall survival in diffuse large B-cell lymphomas except for CNS lymphoma cases and Kim *et al*. study.

Study name	Statistics for each study				
	Hazard ratio	Lower limit	Upper limit	Z-Value	p-Value
F-R C	3.448	1.583	7.510	3.117	0.002
Pham-Ledard A	2.940	1.182	7.313	2.320	0.020
	**3.224**	**1.784**	**5.826**	**3.876**	**0.000**

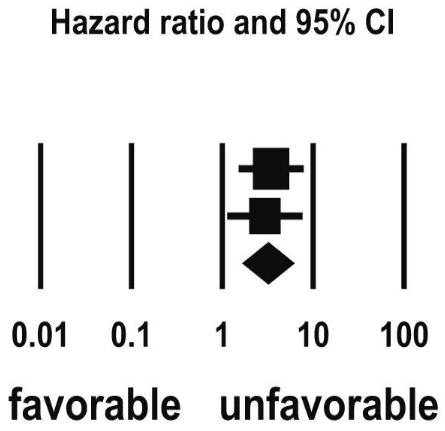

Supplement: Supplementary file 1 — Supplementary Information [file 41598_2017_1998_MOESM1_ESM.pdf]
